# Quantification in bone SPECT/CT

**DOI:** 10.3389/fnume.2026.1941906

**Published:** 2026-09-01

**Authors:** Jovana Zivanovic, Jules Zhang-Yin

**Affiliations:** 1 Centre for Nuclear Medicine and PET, University Clinical Centre of Serbia, Belgrade, Serbia; 2 Clinique Sud Luxembourg, Vivalia, Arlon, Belgium

**Keywords:** bone metastases, bone scintigraphy, bone SPECT/CT, quantitative spect/ct, skeletal imaging, standardized uptake value (SUV)

## Abstract

**Background:**

Quantitative bone SPECT/CT enables objective assessment of skeletal radiotracer uptake using standardised uptake values (SUVs) and related parameters. Advances in reconstruction, correction methods, calibration and detector technology have improved measurement reliability and expanded interest in oncological and benign skeletal applications. This review evaluates current clinical applications, technical limitations and future directions of quantitative bone SPECT/CT.

**Methods:**

PubMed was searched for English-language full-text human studies published from January 2015 to March 2025 using a reproducible Boolean strategy focused on quantitative or semi-quantitative bone SPECT/CT. Screening followed PRISMA 2020 principles. Thirteen primary studies were included in the qualitative synthesis; reviews, case reports, guidelines, position papers and other supporting publications were used only for narrative context.

**Results:**

Quantitative bone SPECT/CT showed potential value for differentiating benign from malignant bone lesions, comparison with 18F-NaF PET/CT, longitudinal response assessment and prognostic evaluation before bone-targeted radionuclide therapy. Potential applications were also identified in condylar hyperplasia, fibrous dysplasia, vertebral compression fractures and degenerative joint disease. However, most studies were retrospective, single-centre and technically heterogeneous. Reported SUV thresholds were influenced by tumour type, lesion phenotype, uptake time, acquisition/reconstruction parameters, calibration, segmentation and recovery-coefficient methods, limiting transferability.

**Conclusions:**

Quantitative bone SPECT/CT is a promising technique for objective assessment of bone metabolism, but current evidence supports cautious use as an adjunct to morphology and clinical context. Prospective multicentre studies with harmonised protocols, risk-of-bias control and patient-relevant endpoints are required. Artificial intelligence, radiomics, advanced detectors and theranostics should currently be considered research opportunities rather than established applications.

## Introduction

Bone SPECT/CT is a method that is used to assess lesions in the skeletal system. Bone imaging began with planar bone scintigraphy that allowed the analysis of the whole skeletal system at once. This was, and still is, especially important in the assessment of widespread osteoblastic bone metastases. It is also useful for assessing metabolic bony disease, primary bone tumours and prosthetic joints. Bone SPECT/CT is an additional method that allows physicians to visualize bone lesions in greater detail. This can help in equivocal findings, or when the exact location of the lesion is unclear, commonly used for spinal lesions.

Bone scintigraphy and SPECT/CT have traditionally relied on qualitative or semi-quantitative interpretation of radiotracer uptake patterns. Although visual assessment remains valuable in routine clinical practice, it is inherently subjective and can be influenced by observer bias. With the introduction of quantitative SPECT/CT, objective uptake parameters such as standardised uptake values (SUVs) that measure radiotracer distribution in a similar manner to PET/CT imaging can now be calculated (1). This in turn has created new opportunities for lesion characterisation, assessment of disease burden, treatment monitoring and response evaluation in both malignant and benign skeletal disorders (1).

Recent advances in detector technology, image reconstruction algorithms, attenuation and scatter correction techniques, and system calibration have significantly improved the accuracy and reproducibility of quantitative SPECT/CT measurements (2–4). Furthermore, the widespread availability of technetium-99m-labelled radiopharmaceuticals and SPECT/CT systems makes quantitative SPECT/CT a potentially more accessible and cost-effective alternative to PET/CT in selected clinical settings (1, 4). As a result, quantitative bone SPECT/CT has emerged as a promising imaging modality with applications extending beyond the detection of skeletal abnormalities towards objective assessment of bone metabolism and disease activity.

Despite these advances, several challenges remain before quantitative SPECT/CT can be fully integrated into routine clinical practice. Variability in acquisition protocols, reconstruction methods, calibration procedures and scanner design may influence quantitative measurements and limit standardisation between institutions (3–6). Consequently, ongoing technological developments and harmonisation efforts are required to ensure accurate and reproducible quantification across different imaging platforms.

This review aimed to evaluate the current clinical applications, technical limitations, and future directions of quantitative bone SPECT/CT.

## Materials and methods

A systematic literature search was performed using the PubMed database to identify studies investigating quantification in bone SPECT/CT and covered articles published between January 2015 and March 2025. The complete PubMed search strategy was: (“SPECT/CT” OR “SPECT CT” OR “single photon emission computed tomography”) AND (“bone” OR “skeletal” OR “bone scintigraphy” OR “bone scan”) AND (“quantification” OR “quantitative” OR “semiquantitative” OR “standardized uptake value” OR “standardised uptake value” OR “SUV” OR “uptake value”). The search was restricted to human studies published in English and available in full text. Reference lists of eligible articles and relevant reviews were additionally screened to identify potentially relevant publications not retrieved by the database search. The full-text and English-language restrictions were retained for feasibility but were considered potential sources of availability and language bias.

Studies were included if they met all the following criteria: (1) investigated human participants undergoing bone SPECT/CT; (2) included quantitative or semi-quantitative analysis of SPECT/CT data; (3) evaluated an oncological or benign skeletal application; (4) reported original clinical or clinically relevant methodological data; (5) were published between January 2015 and March 2025; and (6) were available as full-text articles in English.

On the other hand, studies were excluded if they investigated planar bone scintigraphy without SPECT/CT quantification, PET/CT without corresponding SPECT/CT analysis, non-skeletal applications, animal studies, phantom-only studies without a clinical component, conference abstracts, editorials, or non-English publications. Reviews, systematic reviews, meta-analyses, guidelines, position papers and case reports were excluded from the primary-study evidence base but could be cited as supporting literature when they provided relevant clinical context, technical background or consensus recommendations.

Primary research studies meeting these criteria constituted the evidence base for qualitative synthesis. The study-selection process was performed by the two authors through title/abstract screening and full-text assessment. Disagreements were resolved by discussion. Because the search was performed in a single bibliographic database, no automated duplicate-removal step was applied before title and abstract screening; however, duplicate or overlapping data identified during full-text assessment were excluded and recorded under the appropriate exclusion category. No protocol was registered.

**Figure 1:**
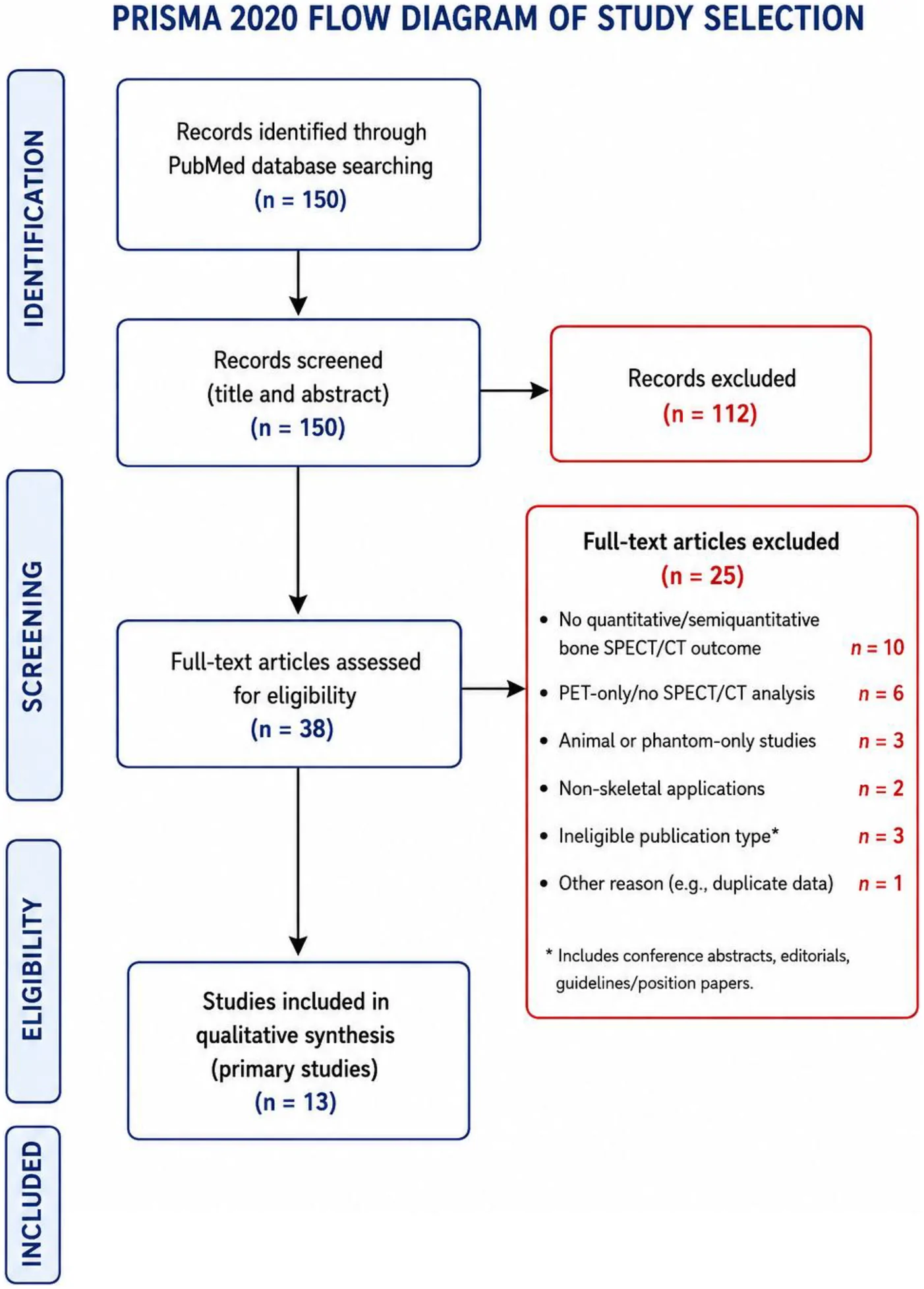
PRISMA 2020 flow diagram of the study-selection process. Thirteen primary studies were included in the qualitative synthesis. Reviews, guidelines, position papers, case reports and technical publications were used only as supporting literature when appropriate.

A total of 150 records were identified through PubMed. Following title and abstract screening, 112 records were excluded and 38 articles underwent full-text assessment. Twenty-five full-text articles were excluded: 10 due to the absence of quantitative or semi-quantitative bone SPECT/CT outcome data, 6 due to PET-only or no SPECT/CT analysis, 3 due to animal or phantom-only studies, 2 due to non-skeletal applications, 3 due to ineligible publication types including conference abstracts, editorials, guidelines or position papers, and 1 due to other reasons including duplicate data. Thirteen primary studies were included in the qualitative synthesis. The study-selection process is summarised in the PRISMA 2020 flow diagram (Figure 1).

Data extracted from the selected studies included study design, patient population, tracer and imaging modality, quantitative endpoint(s), acquisition and reconstruction information, calibration and correction methods, segmentation approach, reference standard or outcome measure, and principal clinical findings. Particular attention was paid to methodological features affecting the transferability of SUV thresholds, including uptake time, handling of injected activity, attenuation and scatter correction, reconstruction settings, voxel or matrix size, segmentation strategy, calibration procedures, and recovery-coefficient methodology. As these items were reported inconsistently across the available literature, any missing or insufficiently reported technical details are explicitly marked in Table 1 rather than being inferred. Due to heterogeneity in study design, methodology and outcomes, a qualitative narrative synthesis was performed rather than a formal meta-analysis.

**Table 1:** Summary of the thirteen primary clinical and methodological studies.

Category	Study	Design/evidence type	Sample size	Tracer/comparison	Endpoint(s)	Methodological details relevant to transferability	Key findings	Main limitations
Bone metastases/oncology	Du et al. (10)	Retrospective derivation plus prospective validation	Part I: 167 patients, 396 lesions; Part II: 49 patients, 49 solitary lesions	Bone SPECT/CT, diphosphonate tracer	SUVmax	Lesion-level SUVmax threshold; technical transferability depends on uptake time, injected-activity measurement, reconstruction, calibration, segmentation and RC details; single-centre threshold requires local validation.	Malignant lesions showed higher SUVmax than benign lesions; SUVmax >18.07 identified malignancy with reported prospective sensitivity 82.35%, specificity 93.75% and accuracy 89.80%.	Threshold should not be generalised across tumour types or camera/reconstruction protocols without validation.
Bone metastases/oncology	Lin et al. (11)	Retrospective cohort	115 patients with lung adenocarcinoma	Bone SPECT/CT	SUVmax	Lung-cancer-specific lesion-level SUVmax analysis; osteoblastic, mixed, osteolytic and CT-negative lesions analysed separately; cross-vendor calibration and RC transferability not established.	Metastases had higher SUVmax than benign lesions; SUVmax 11.10 distinguished benign from malignant lesions; SUVmax 8.135 helped separate CT-negative metastases from normal vertebrae.	Single-centre and disease-specific; findings should not be treated as universal skeletal thresholds.
Bone metastases/oncology	Arvola et al. (12)	Comparative imaging study	26 patients	99mTc-HDP SPECT/CT compared with 18F-NaF PET/CT	SUVmax, SUVmean	Direct SPECT/PET quantitative comparison; interpretation should consider lesion-level vs patient-level analysis and limits of agreement, not correlation alone.	SPECT/CT-derived SUVs correlated with 18F-NaF PET/CT SUVs in breast and prostate cancer bone metastases.	Correlation does not prove interchangeability; small cohort.
Bone metastases/oncology	Beck et al. (13)	Longitudinal observational study	44 patients	99mTc-DPD SPECT/CT	SUVmax, SUVmean	Serial quantitative assessment; reproducibility assessed; outcome impact not directly tested.	Quantitative longitudinal analysis showed higher interobserver reproducibility than visual assessment.	Supports reproducibility and feasibility more than direct patient-outcome benefit.
Bone metastases/oncology	Gherghe et al. (14)	Retrospective response-assessment study	36 patients	Bone SPECT/CT in metastatic breast cancer	SUVmax, summed SUVmax, TLA	Response evaluated using quantitative change metrics; segmentation and thresholding approach affect TLA and summed SUV endpoints.	Quantitative assessment identified response patterns not always concordant with qualitative interpretation.	Retrospective, single-centre; response thresholds require prospective validation.
Bone metastases/oncology/therapy	Dittmann et al. (15)	Prospective/observational prognostic study before 223Ra therapy	44 patients	Quantitative bone scan SPECT/CT before 223Ra	Skeletal tumour burden, SUV parameters	Baseline burden metrics; outcome association with survival; not a management-impact trial.	Quantitative skeletal tumour burden and uptake parameters were associated with outcomes before 223Ra therapy.	Prognostic association does not prove improved treatment selection.
Fibrous dysplasia	Zhang et al. (18)	Retrospective clinical study	21 patients	99mTc-MDP SPECT/CT	Relative tracer uptake/metabolic activity	Hybrid SPECT/CT used for lesion characterisation; absolute SUV thresholding not the main endpoint.	SPECT/CT improved lesion characterisation and helped differentiate fibrous dysplasia from metastases when scintigraphy was positive.	Small cohort; quantitative method not directly transferable to SUV thresholds.
Fibrous dysplasia	Jreige et al. (19)	Retrospective observational study	7 patients	99mTc-diphosphonate planar bone scan plus quantitative SPECT/CT	SUVmax, SUVmean, skeletal burden score	Combined planar skeletal-burden score with lesion SUV metrics; small-volume disease and segmentation affect estimates.	Quantitative parameters correlated with biological bone turnover markers.	Very small sample; exploratory biomarker association.
Normal reference values	Qi et al. (20)	Retrospective reference-value study	221 patients	99mTc-MDP quantitative SPECT/CT	SUVmax in normal vertebrae	Normal vertebral VOIs; sex and vertebral-level effects reported; reference values depend on local acquisition, reconstruction and calibration.	Established normal vertebral SUV values and observed sex-related differences.	Reference ranges may be scanner- and population-specific.
Normalisation/methodology	Damian et al. (21)	Retrospective methodological study	119 patients selected from lumbar-spine SPECT/CT scans	99mTc-HDP quantitative SPECT/CT	SUVmean/SUVmax, activity concentration, CT-derived bone density	Body weight, age and CT-derived bone density analysed as normalisation factors; illustrates patient-specific effects on uptake.	Standard body-weight SUV may overestimate uptake in heavier patients; age and bone density significantly influenced vertebral uptake.	In-sample model; residual interindividual variability remained high.
Degenerative/orthopaedic applications	An et al. (22)	Comparative clinical study	52 patients	Bone scintigraphy versus bone SPECT/CT	SUV/uptake grading by knee compartment	Compartment-based assessment; planar grades compared with SPECT/CT findings; calibration details relevant if SUV thresholds are extrapolated.	SPECT/CT detected and localised more uptake abnormalities than planar scintigraphy in knee assessment.	Comparative imaging value shown; clinical outcome impact not established.
Vertebral compression fractures	Wang et al. (28)	Retrospective diagnostic study	34 VCF patients with 57 fresh VCFs, 57 adjacent normal vertebrae and 19 old VCFs; 52 controls	Quantitative radionuclide bone SPECT/CT	SUVmax	Fresh versus old fracture and normal/control vertebrae; threshold depends on fracture age, analysis software and acquisition/reconstruction protocol.	Fresh fractures showed higher SUVmax; SUVmax cut-off 9.925 differentiated fresh from old fractures.	Retrospective; threshold differs from prior reports and requires external validation.
Methodological reconstruction	Kangasmaa et al. (2)	Phantom plus clinical methodological study	14 patients plus phantom simulations	Quantitative bone SPECT/CT reconstruction with anatomical-prior methods	SUVmean	Compared OSEM with Bayesian/anatomical-prior reconstruction; CT-emission misregistration and reconstruction model affect quantitative values.	Bayesian/anatomical-prior reconstruction produced SUVs closer to true activity than OSEM in simulations and higher clinical SUVs.	Methodological study; reconstruction-dependent SUV values are not directly comparable with OSEM thresholds.

### Risk of bias assessment

A structured study-by-study risk-of-bias appraisal of the 13 primary studies included in the qualitative synthesis was performed using design-appropriate domains rather than a single disease-specific tool, because the included studies were heterogeneous in design and objectives (Table 2). The assessment considered potential bias arising from participant selection, study design, measurement of quantitative SPECT/CT parameters, outcome assessment, confounding factors, completeness of reported data and appropriateness of statistical analysis, where applicable. For methodological imaging studies, additional consideration was given to the reference standard, reproducibility of quantitative measurements and potential sources of technical measurement bias.

**Table 2:** Structured risk-of-bias appraisal of the 13 primary studies included in the qualitative synthesis.

Study	Participant selection	Quantitative/index-test methodology	Reference standard or outcome	Confounding/external validation	Overall judgement	Main reason
Du et al. (10)	Moderate	Moderate	Moderate	Moderate	Moderate	Retrospective derivation with prospective validation, but single-centre threshold and technical transferability remain limited.
Lin et al. (11)	Moderate	Moderate	Moderate	High	Moderate to high	Disease-specific retrospective cohort; thresholds need external validation.
Arvola et al. (12)	Moderate	Moderate	Moderate	High	Moderate	Small comparative imaging cohort; correlation does not establish interchangeability.
Beck et al. (13)	Moderate	Low to moderate	Moderate	Moderate	Moderate	Good reproducibility focus, but limited clinical-outcome assessment.
Gherghe et al. (14)	Moderate	Moderate	Moderate	High	Moderate to high	Retrospective response assessment with heterogeneous disease and treatment context.
Dittmann et al. (15)	Moderate	Moderate	Moderate	Moderate	Moderate	Prognostic association study; management impact not directly tested.
Zhang et al. (18)	Moderate	Moderate	Moderate	High	Moderate to high	Small retrospective benign-disease cohort; limited external validation.
Jreige et al. (19)	High	Moderate	Moderate	High	High	Very small cohort; exploratory correlation with biomarkers.
Qi et al. (20)	Moderate	Moderate	Low to moderate	Moderate	Moderate	Reference-value study; population and scanner dependence limit generalisability.
Damian et al. (21)	Moderate	Moderate	Low to moderate	Moderate	Moderate	Robust methodological question but in-sample normalisation model and residual patient effects.
An et al. (22)	Moderate	Moderate	Moderate	High	Moderate to high	Comparative imaging findings without definitive outcome validation.
Wang et al. (28)	Moderate	Moderate	Moderate	High	Moderate to high	Retrospective threshold study; cut-off differs across reports and requires external validation.
Kangasmaa et al. (2)	Low to moderate	Low	Moderate	Moderate	Moderate	Strong technical design but phantom/methodological focus limits direct clinical applicability.

Publications used exclusively as supporting literature, including reviews, guidelines, position papers and case reports, were not included in the risk-of-bias assessment because they did not form part of the primary-study qualitative synthesis.

### Additional technical principles of quantitative bone SPECT/CT

Quantification in SPECT/CT requires accurate conversion of detected counts into absolute activity concentration. SUV is usually calculated as activity concentration within a volume of interest divided by injected activity normalised to body weight. The most commonly reported parameters are SUVmax, SUVmean and SUVpeak. SUVmax is simple and sensitive to focal uptake, but it is affected by image noise because it depends on a single voxel; SUVmean is more stable but depends strongly on segmentation; and SUVpeak, which averages uptake over a small fixed volume around the hottest region, may offer a useful compromise for follow-up studies and multicentre research (7–9).

Reliable quantification depends on a complete correction and calibration chain. CT-based attenuation correction compensates for photon loss within tissues; scatter correction reduces contamination from deflected photons; resolution recovery or point-spread-function modelling partially compensates for collimator-detector blurring; and dead-time correction may be required in high-count situations. Cross-calibration between the dose calibrator and gamma camera is essential, usually through phantom-based procedures, and recovery coefficients should be characterised for different lesion sizes because partial-volume effects remain a major source of underestimation in small lesions (3, 7).

### Current state

Until recently, the quantification of nuclear medicine data was only available using PET/CT methods. The spatial resolution and iterative reconstruction algorithms of PET/CT systems allowed for the interpretation of data (1). Due to the development of new technology for quantitative metrics, the same quantification methods are now applicable to SPECT/CT methods too. This in turn resulted in the ability to assess a larger range of radiopharmaceuticals that are highly available at a lower cost than PET radiopharmaceuticals (1). Quantification in bone SPECT/CT refers to the measurement of radiotracer uptake using objective numerical parameters such as SUV, allowing bone metabolism and osteoblastic activity to be assessed quantitatively rather than by visual interpretation alone. The main application for quantification of bone SPECT/CT is currently in the evaluation of bony metastases due to malignant disease, namely prostate cancer (1). It is also of significance in other malignant tumours such as breast, lung, bladder, renal cell carcinoma and melanoma (10). The relative incidence of bone metastases has been reported to be 65%–75% in prostate cancer, 65%–75% in breast cancer, 30%–40% in lung cancer, 40% in bladder cancer, 20%–25% in renal cell carcinoma and 14%–45% in melanoma (11). There are other applications for bone SPECT/CT for benign disease such as bone and fibrous dysplasia.

Several studies have shown that quantitative assessments of bone SPECT/CT examinations are possible. Arvola et al. demonstrated that standardised uptake values (SUVs) calculated using SPECT/CT studies using 99mTc-HDP were closely associated to those using 18F-NaF PET/CT (12). The addition of SPECT/CT to whole body planar scintigraphy has gradually improved the limitations of specificity (10). In one study, it was shown that 62% of the lesions that were deemed equivocal on WBS could be identified by SPECT while 85%–92% of lesions undetermined by SPECT were then identified by SPECT/CT (3). Yamane et al. demonstrated that SUVmax calculated using SPECT/CT has good reproducibility and can be used as a reliable indicator in patient management (10). Furthermore, several studies have shown that SUVmax of bone quantitative SPECT/CT is helpful to differentiate between benign and malignant bone lesions in malignant disease (10). Du et al. demonstrated that the SUVmax of malignant lesions was significantly higher than that of benign lesions and normal vertebrae, while no statistically significant difference was observed between the SUVmax values of different types of bone metastases (10). The SUVmax of fractures was much higher than the SUVmax of non-fracture lesions (10). This study calculated that a SUVmax over 18.07 could be used as a diagnostic criterion of malignancy with a sensitivity of 82.35%, specificity of 93.75% and accuracy of 89.80% (10).

A study that characterised metastatic bone lesions in lung cancer also demonstrated that metastatic lesions had a higher SUVmax than benign lesions and fractures (11). In this study, it was also shown that a cutoff SUVmax of 11.10 was accurate in distinguishing between benign and malignant lesions, with a sensitivity of 87.70% and a specificity of 80.71% (11). However, this study demonstrated that osteoblastic and mixed lesions had a significantly greater SUVmax than osteolytic and CT-negative lesions (11). For distinguishing between CT-negative bone metastases from normal vertebrae, a SUVmax at a cutoff value of 8.135 was identified (11).

The SUV thresholds reported in individual studies should not be interpreted as universal or directly transferable diagnostic cut-offs. Quantitative values are influenced by tumour type and lesion phenotype, but also by technical factors including administered activity, uptake time, acquisition parameters, scanner calibration, reconstruction algorithms, attenuation and scatter correction, segmentation methodology, partial-volume effects and recovery-coefficient methods. Consequently, thresholds derived from a single scanner, reconstruction protocol or patient cohort require independent validation and appropriate harmonisation before being applied at other institutions. Quantitative SPECT/CT should therefore currently be considered an adjunct to CT morphology, clinical information and longitudinal imaging rather than a stand-alone diagnostic test (1, 7, 10, 11).

Quantitative SPECT/CT also has potential for treatment-response assessment. Beck et al. demonstrated that longitudinal quantitative SPECT/CT analysis of bone metabolism produced almost perfect interobserver agreement, while visual analysis produced lower agreement. Gherghe et al. showed that quantitative SPECT/CT can identify response patterns in metastatic breast cancer that are not always concordant with qualitative interpretation. Beyond single-lesion SUVmax, volumetric metrics such as total lesion activity and skeletal tumour burden may provide global markers of disease extent, analogous to metabolic tumour volume and total lesion glycolysis in FDG PET/CT. Such approaches may be particularly relevant for radiopharmaceutical therapy and for predicting outcomes before or during 223Ra treatment (13–15).

Another important indication for bone scintigraphy is the evaluation of benign bone lesions. Mandibular condylar hyperplasia is a complex development deformity that can result in asymmetries in the hyperplastic condyle (16). It causes facial asymmetry and is associated with pain and mandibular dysfunction (16). Bone SPECT has a role in detecting the growth rate of this disorder (16). Moreover, this method can detect uptake differences between the right and the left. An uptake difference of around 10% or more on the ipsilateral side of condylar hyperplasia is used as evidence that there is actively growing disease, which is important in aiding physicians for treatment selection (16). Treatment options include partial condylectomy for active condylar hyperplasia and observation for inactive condylar hyperplasia (16). With the addition of CT to SPECT/CT, condylar dimensions and other bony changes can simultaneously be assessed (16). In one particular case report, the bone SPECT showed that there was an asymmetric radionuclide uptake between the left and right condyles (16). Calculated uptake was 55% on the right and 45% on the left, but the 10% difference was on the contralateral side to the condylar hyperplasia, an indication of inactive growth (16). On planar scintigraphy, asymmetric radionuclide uptake was not seen (16).

When it comes to unilateral condylar hyperplasia, bone SPECT can be used to assess the condylar bone activity on the affected side (17). One study calculated that the relative uptake difference between the affected and unaffected side was about 55% (17). A few further studies showed similar cutoff values, ranging between 54%–56% (17). Hamed et al. used a combined quantification method to diagnose unilateral condylar hyperplasia (17). A patient was only considered to have unilateral condylar hyperplasia if all of the following conditions were met: a relative activity uptake of ≥55%, an absolute condylar uptake of >10%, a radiotracer count ratio of >1.10, a condyle-to-clivus ratio of >1.44 and an enlarged condyle with sclerosis and ground glass haziness on the CT scan (17).

Fibrous dysplasia is a benign bone disorder in which normal bone is replaced by immature fibro-osseus tissue (6). It is a rare disorder (18). Fibrous dysplasia has demonstrated increased radionuclide uptake on bone scintigraphy (18). This is important for differentiating between benign bone disease and potential metastases, leading to false positive results (18). In diagnosing fibrous dysplasia, SPECT/CT hybrid imaging provides useful anatomical information that can help differentiate between these types of lesions (18). One study analysed 21 cases out of which 18 cases demonstrated moderate and high metabolism of MDP (18). Biopsy or surgery was performed on 13 out of the 18 positive patients, and all pathological results were reported as fibrous dysplasia (18).

Another study stated that the skeletal burden score that can be derived from a bone scan using MDP is a valuable instrument for measuring the burden of fibrous dysplasia on the whole skeleton (19). Its limitation is that it is based on two-dimensional imaging (19). Van der Bruggen et al. used Na18F PET/CT for quantification of these lesions which displayed a strong correlation between the radiotracer and biological bone turnover markers (19). This sparked the question of whether this technique could be used as treatment follow-up, which in turn led to the use of SPECT/CT quantification using DPD for treatment follow up (19). The study used a combination of planar and absolute SPECT/CT quantification in the following way: the skeletal burden score was multiplied by the average SUVmax and SUVmean values of the fibrous dysplasia lesions which produced disease corrected SUVmax and SUVmean values (19). The correlation between the two values provided a more accurate representation of the biological activity of fibrous dysplasia (19).

Bone scintigraphy can be used for assessing normal bone structure. A study that investigated establishing baseline values for SUVmax in vertebral regions in males and females showed that SUVmax of normal cervical, thoracic and lumbar vertebrae in male patients was significantly higher than in female patients (20). The SUVmax of all three vertebral regions in male patients showed statistical differences (20).

Patient-specific SUV normalisation and normal reference values are increasingly recognised as important issues in bone SPECT/CT. Diphosphonate tracers do not accumulate in adipose tissue in the same way as some PET tracers, and normal bone uptake is influenced by age, sex, body habitus, vertebral level, bone density and tracer clearance. Damian et al. investigated whether body weight, age and CT-derived bone density could improve normalisation of 99mTc-HDP bone SPECT/CT. They found that standard body-weight SUV may overestimate uptake in heavier patients and that age and bone density significantly influence normal vertebral uptake (20, 21).

This issue is clinically important because many proposed thresholds for metastasis, fracture activity or degenerative disease are based on SUVmax. If background bone uptake varies widely between patients, a single universal cut-off may be misleading. Future quantitative bone SPECT/CT may therefore require patient-specific correction models incorporating lean body mass, bone mineral density, renal function, hydration status, tracer clearance and CT-derived bone density (20, 21).

One study demonstrated the added value of SPECT/CT in assessing knee joints when compared to bone scintigraphy (22). The study evaluated the degree of uptake in each of the following knee regions: medial femoral, lateral femoral, medial tibial, lateral tibial and patellar (22). Planar images and SPECT/CT were evaluated using visual scoring grades 0 to 2 and quantitative analyses for SPECT/CT using uptake counts for planar image and SUVs (22). The results showed that the uptake grades assessed visually on the planar images differed significantly from all of the uptake grades on SPECT/CT images in all areas of the knee (22). It was also shown that more lesions can be identified using SPECT/CT when compared to bone scintigraphy (22).

In painful hip and knee arthroplasty, SPECT/CT can help differentiate loosening, malalignment, overload and infection by integrating uptake patterns with CT morphology. This is particularly useful when radiographs are inconclusive or MRI is limited by metallic artefacts. Quantification may make these interpretations more objective and could facilitate follow-up after revision surgery or conservative treatment (23, 24).

Limitations of these studies still exist. Bone lesions in SPECT/CT may show focal abnormal radioactivity concentration on SPECT images in the absence of an identifiable lesion on CT (2). This is especially important in solitary lesions which often further examination using other imaging methods which may be more expensive (2).

When it comes to the assessment of growth activity in condylar hyperplasia using bone scintigraphy, degenerative and inflammatory arthropathies of the temporomandibular joint can complicate the evaluation since these conditions demonstrate an increased uptake of MDP (4).

Positron emission tomography/computed tomography (PET/CT), particularly using 18F-NaF, is often regarded as the reference standard for quantitative skeletal imaging due to its high spatial resolution, superior sensitivity and established quantitative capabilities. Quantification in PET/CT has been routinely used for many years, whereas quantitative SPECT/CT has only recently become clinically feasible through advances in detector technology, image correction methods and reconstruction algorithms (1).

Several studies have demonstrated a strong correlation between quantitative parameters obtained from bone SPECT/CT and those derived from PET/CT. Arvola et al. reported that SUV measurements obtained using 99mTc-HDP SPECT/CT closely correlated with those obtained using ^18^F-NaF PET/CT, suggesting that quantitative SPECT/CT may provide clinically meaningful information comparable to PET/CT in selected applications (1). However, PET/CT generally maintains advantages in spatial resolution, image quality and quantitative accuracy.

The quantitative comparison between 99mTc-HDP SPECT/CT and 18F-NaF PET/CT is particularly important because it suggests that SPECT/CT-derived SUVs may provide clinically meaningful information in centres where PET/CT is unavailable or not justified, while still acknowledging that PET/CT usually maintains advantages in spatial resolution, image quality and quantitative accuracy (12).

Despite these advantages, PET/CT remains less accessible than SPECT/CT in many institutions due to higher equipment costs, limited availability and the need for PET-specific radiopharmaceuticals. In contrast, technetium-99m-labelled tracers are widely available and relatively inexpensive, making quantitative SPECT/CT an attractive alternative for skeletal imaging (1). Consequently, while quantitative SPECT/CT is unlikely to replace PET/CT in all clinical scenarios, it may provide a cost-effective and widely accessible method for quantitative assessment of bone metabolism and disease activity.

The available clinical evidence should, however, be interpreted cautiously. Much of the literature consists of retrospective, observational and single-centre studies with relatively small and heterogeneous patient populations. In addition, differences in radiopharmaceuticals, acquisition protocols, reconstruction algorithms, calibration procedures, segmentation methods and quantitative endpoints limit direct comparison between studies. Although these investigations demonstrate the technical feasibility of quantitative bone SPECT/CT and identify potentially useful associations between quantitative parameters and clinical or imaging findings, evidence that quantification directly improves diagnostic accuracy, treatment decisions or patient outcomes remains limited. Prospective, multicentre studies using harmonised imaging protocols and clinically relevant endpoints are therefore required to establish the incremental clinical value of quantitative bone SPECT/CT.

### Technical challenges

Quantification in nuclear medicine is heavily reliant on the quality of the images produced and their ability to be turned into data. Therefore, technical aspects are crucial to the overall results of quantification. The main technical aspects that should be considered when performing quantitative analyses are reconstruction and standardisation of data. The conventional reconstruction method used in SPECT is the ordered subsets expectation maximization (OSEM) reconstruction method. The OSEM method makes assumptions about how the image should appear before the reconstruction. However, OSEM reconstruction has several limitations, including image noise, convergence-related bias and reduced quantitative accuracy in small lesions. Despite that, it is widely accepted as the standard for SPECT. One study compared Bayesian reconstruction methods that used anatomical prior information from CT (AMAP-S and AMAP-R) to a Bayesian reconstruction method without anatomical information (RDP used in PET) to OSEM reconstruction (2). The results of the initial study using artificial lesions showed that the three Bayesian methods provided SUVmean values which were closer to the true SUV value than OSEM for all the lesions studied (2). Likewise, in the actual clinical patient studies, the Bayesian methods displayed higher SUVs than OSEM, and the difference between them was statistically significant (2). When reconstruction is performed with an anatomical prior, it is susceptible to misalignment between the anatomical and emission images at a far greater extent than without anatomical prior (2). Bayesian reconstruction up until recently did not have a place in regular clinical practice (2). However, with improving reconstruction using RDP in PET it is becoming more endorsed (2). Since Bayesian reconstruction with anatomical prior showed better results than OSEM reconstruction, it could be seen as a suitable alternative for bone SPECT/CT reconstruction in the future (2).

In addition to reconstruction-related variability, recent developments in SPECT hardware have introduced further challenges regarding the standardisation of quantitative measurements.

Recent advances in SPECT/CT hardware have led to the development of cadmium-zinc-telluride (CZT) detector systems arranged in a ring geometry configuration. Unlike conventional dual-head Anger cameras that rotate around the patient, ring-configured systems consist of multiple stationary or semi-stationary detector modules positioned around the patient in a 360° arrangement. This design substantially increases photon sensitivity and count statistics by enabling a greater proportion of emitted photons to be detected simultaneously (3, 5, 6). Improved sensitivity may allow shorter acquisition times, lower administered activities and improved image quality, all of which are advantageous for quantitative imaging (5, 6).

The introduction of ring geometry has also been associated with improvements in spatial resolution and energy resolution when combined with CZT detector technology. Enhanced energy resolution improves scatter correction and may contribute to more accurate quantification of radiotracer uptake, while improved sensitivity increases the reliability of quantitative parameters such as SUVmax and SUVmean (3, 6). Furthermore, these systems have demonstrated the potential to perform dynamic SPECT acquisitions, which may enable kinetic analysis of tracer uptake in the future (3, 6).

Despite these advantages, several technical challenges remain. Ring-configured CZT systems differ substantially from conventional SPECT cameras in detector design, acquisition protocols and reconstruction algorithms. As a result, quantitative measurements obtained on different systems may not be directly comparable (3, 5). The lack of standardised calibration procedures and harmonised reconstruction settings may introduce variability in SUV measurements between institutions and scanner vendors (5). In addition, the evidence supporting routine clinical implementation of ring-configured systems remains relatively limited, and further multicentre validation studies are required before quantitative metrics derived from these systems can be universally adopted (5, 6).

Consequently, although ring-configured CZT SPECT/CT systems represent a significant technological advancement with the potential to improve quantitative bone imaging, ongoing efforts toward standardisation, validation and protocol harmonisation are necessary to ensure reproducible and clinically reliable quantitative measurements (3, 5, 6).

Additional technical challenges affecting quantitative bone SPECT/CT include inter-scanner variability and the lack of harmonised quantitative protocols. Quantitative measurements may differ between imaging systems due to variations in detector technology, collimator design, acquisition parameters, reconstruction algorithms and calibration procedures (3, 5, 6). Consequently, SUV measurements obtained on one scanner may not be directly comparable to those obtained on another, limiting the reproducibility of quantitative data across institutions (3, 5).

Furthermore, unlike PET/CT, quantitative SPECT/CT currently lacks universally adopted harmonisation standards. Although recent technological advances have improved the accuracy and reliability of quantitative SPECT/CT, substantial variability in imaging protocols and reconstruction settings persists between centres (3, 5). Therefore, the development of standardised acquisition, reconstruction and quantification protocols remains an important requirement for the wider clinical implementation of quantitative bone SPECT/CT and for ensuring reproducible quantitative measurements across different imaging platforms (3, 5, 6).

Standard operating procedures should therefore include phantom calibration, quality control after software or hardware updates, recovery coefficient assessment, harmonised display scales and clear reporting of acquisition and reconstruction parameters. Clinical reports should integrate SUV measurements with CT morphology, lesion distribution, prior imaging and clinical information. Quantification should support clinical reasoning rather than replace it (7).

The lack of standardised quantitative protocols may also contribute to variability in the reported diagnostic thresholds used in clinical practice. This is particularly evident in the assessment of vertebral compression fractures, where quantitative SPECT/CT has been investigated as a tool for differentiating acute from chronic fractures.

Amongst benign lesions, bone scintigraphy plays a significant role in assessing potential vertebral compression fractures. Vertebral fractures can be caused by trauma, but they are more commonly related to insufficiency and osteoporosis (6). Osteoporosis affects more than 200 million people worldwide, and it has been estimated that 30%–50% of women and 20%–30% of men may experience a vertebral compression fracture (6). The difficulty in assessing vertebral compression fractures is determining whether a fracture is fresh or old (6). Wang et al. evaluated quantitative bone SPECT/CT in 34 patients with vertebral compression fracture, including 57 fresh vertebral compression fractures, 57 adjacent normal vertebrae, 19 old vertebral compression fractures and 52 controls. Fresh fractures had significantly higher SUVmax values than old fractures and normal vertebrae, and an SUVmax cut-off value of 9.925 was identified for diagnosing fresh vertebral compression fractures (28). Wang et al. also noted that a previous quantitative study reported a higher SUVmax cut-off of 18.5, which may reflect differences in patient characteristics, disease course, software and quantitative methodology (28).

### Future direction

There are many ways in which SPECT/CT is yet to help improve diagnostic accuracy of bone scintigraphy. With the introduction of CZT detector-based SPECT/CT cameras, the sensitivity, energy resolution and image contrast of images have significantly improved (6). In the future, we can expect that SPECT/CT designs will benefit from advances in CT technology which will in turn lead to high spatial resolution and lower dose scanning for all body regions (6).

Full-ring 360-degree CZT SPECT/CT systems are particularly relevant to bone imaging. Compared with conventional rotating dual-head cameras, full-ring CZT systems use detector modules arranged around the patient, increasing count sensitivity and improving spatial and energy resolution. The recent EANM position paper on full-ring CZT bone imaging argues that these systems may change the workflow of bone scintigraphy by making routine tomographic bone imaging feasible and reducing dependence on planar whole-body acquisitions (6).

Early implementation and clinical experience reports have described the practical deployment of ring-configured CZT SPECT/CT systems and their comparison with conventional gamma-camera systems. Higher sensitivity may allow shorter acquisitions, lower administered activities, improved image quality and potentially dynamic bone SPECT protocols. Dynamic acquisitions could provide kinetic information about perfusion, blood pool and delayed bone incorporation, moving SPECT beyond static SUV measurement toward functional modelling of bone turnover (5, 6, 25).

However, CZT and full-ring systems also raise new questions. Their detector geometry, fixed or semi-fixed collimators, quality-control procedures and reconstruction algorithms differ from conventional cameras. Multicentre validation, transparent cross-vendor calibration and cost-effectiveness analyses are therefore necessary. SPECT/CT should be considered complementary rather than obsolete in the PET/CT era, particularly because SPECT systems and technetium-labelled tracers remain more widely available in many healthcare settings (26).

Other advances in SPECT/CT technology include the change from bulky photomultiplier tubes to position-sensitive photomultiplier tubes, avalanche photodiodes and silicon photomultipliers (4). Furthermore, new SPECT cameras with dedicated cardiac imaging have been designed (4). The new designs have shorter scanning times, improved image quality, enhanced patient comfort due to reduced claustrophobic effects and a smaller size of the camera in general (4). On the software side, the most noticeable improvements have been through the implementation and commercial availability of reconstruction algorithms, data correction and quantification (4). The use of maximum likelihood expectation maximization (MLEM) and ordered-subsets expectation maximization (OSEM) reconstruction methods in newer SPECT/CT systems has led to improved noise control (4).

Artificial intelligence (AI), radiomics and automated workflows should be regarded as narrative future perspectives in this review rather than as systematically appraised clinical evidence. Radiomics enables the extraction of quantitative data beyond conventional parameters such as SUVmax and SUVmean, allowing more detailed assessment of individual lesion characteristics. When combined with machine-learning algorithms, these features could support automated lesion detection, differentiation between benign and malignant bone lesions and prediction of treatment response, but clinical use will require harmonised multicentre datasets, external validation and transparent reporting of model performance (4). As quantitative SPECT/CT becomes more widely adopted, AI-based tools may improve diagnostic accuracy, reduce observer variability and facilitate more efficient image interpretation (4).

Deep-learning-enhanced acquisition and reconstruction are also emerging. Qi et al. demonstrated that deep-learning-enhanced ultra-fast SPECT/CT can maintain quantitative and clinical performance in patients with suspected malignancy, suggesting that AI may reduce the traditional trade-off between acquisition time and image noise. Future AI tools should be trained on harmonised multicentre datasets and validated externally, because erroneous automated classification could affect cancer staging, biopsy decisions, treatment selection and follow-up (27).

Quantitative SPECT/CT may also have a possible future role in personalised medicine, particularly in theranostic applications within nuclear medicine. The ability to accurately quantify radiotracer uptake can aid physicians in making decisions about patient-specific dosimetry, treatment planning as well as treatment outcome predictions (4, 6). Theranostic applications are usually associated with PET/CT, with the most well-known theranostic pairs being for the treatment of neuroendocrine tumours and prostate cancer. In spite of this, advances in SPECT/CT quantification may facilitate more widespread investigation of personalised treatment strategies using available radiopharmaceuticals. Furthermore, serial quantitative SPECT/CT examinations could enable objective assessment of disease progression and therapeutic response, particularly in patients with metastatic bone disease (4, 6).

Nevertheless, the theranostic role of bone SPECT/CT should be defined carefully. Quantitative diphosphonate uptake reflects osteoblastic reaction and bone turnover, not necessarily viable tumour burden alone. Interpretation should therefore consider lesion phenotype, flare phenomena, symptoms, biochemical markers and complementary imaging. Future prospective trials should evaluate whether quantitative SPECT/CT changes management and improve patient-centred outcomes rather than merely producing more numerical data (14, 15).

CZT detector-based SPECT/CT cameras are innovative, but their exact place in routine practice remains to be defined. Future studies should clarify whether these systems will replace conventional gamma cameras in everyday skeletal imaging or will be used selectively for high-throughput whole-body SPECT/CT, dynamic imaging, theranostics and dosimetry. New reconstruction algorithms may also introduce image-noise amplification and spatial variability of point-spread functions; therefore, their effect on absolute SUV values should be validated before thresholds are transferred between systems (4, 6).

Future trials should move beyond single-centre retrospective threshold studies. Prospective multicentre studies are needed to compare quantitative and qualitative SPECT/CT, evaluate reproducibility, define clinically meaningful SUV changes, and determine whether quantitative imaging improves patient outcomes. Trials should include standardised phantom calibration, prespecified reconstruction settings, central image review and clinically relevant endpoints such as fracture risk, treatment change, pain response, progression-free survival or avoidance of unnecessary biopsy (1, 7).

## Conclusion

Quantitative bone SPECT/CT represents an important technological development in skeletal imaging by enabling objective assessment of radiotracer uptake through parameters such as standardised uptake values. Available studies suggest potential clinical applications in malignant and benign skeletal disease, including the evaluation of bone metastases, vertebral compression fractures, condylar hyperplasia and fibrous dysplasia. Quantitative measurements may complement conventional qualitative interpretation by providing objective information on lesion characteristics and changes over time; however, evidence demonstrating improved clinical outcomes or management compared with conventional assessment remains limited.

Although there are many advantages of SPECT/CT, there are still several technical challenges. Differences in reconstruction methods, scanner design, calibration procedures and acquisition protocols may influence quantitative measurements and limit comparability between institutions. Furthermore, the lack of universally accepted harmonisation standards continues to represent a barrier to the widespread adoption of quantitative SPECT/CT in routine clinical practice.

Ongoing advances in detector technology, particularly CZT-based and ring-geometry systems, may further improve image quality, sensitivity and quantitative performance. Artificial intelligence, radiomics and theranostic applications represent promising areas for future investigation, but they should not be considered established clinical applications on the basis of the current evidence base. Prospective multicentre studies with standardised acquisition, reconstruction, calibration and analysis protocols are needed to determine whether these technological advances translate into improved diagnostic performance, clinical decision-making or patient outcomes. As harmonisation and validation efforts progress, the clinical role of quantitative bone SPECT/CT may become better defined.
